# Antibacterial, Flexible, and Conductive Membrane Based on MWCNTs/Ag Coated Electro-Spun PLA Nanofibrous Scaffolds as Wearable Fabric for Body Motion Sensing

**DOI:** 10.3390/polym12010120

**Published:** 2020-01-05

**Authors:** Lu Gan, Aobo Geng, Ying Wu, Linjie Wang, Xingyu Fang, Lijie Xu, Changtong Mei

**Affiliations:** 1 College of Materials Science and Engineering, Nanjing Forestry University, Nanjing 210037, China; ganlu@njfu.edu.cn (L.G.); abgeng@njfu.edu.cn (A.G.); wuying@njfu.edu.cn (Y.W.); wanglinjie@njfu.edu.cn (L.W.); fangxingyu@njfu.edu.cn (X.F.); 2 College of Biology and the Environment, Nanjing Forestry University, Nanjing 210037, China; xulijie@njfu.edu.cn

**Keywords:** PLA nanofiber, flexible membrane, carbon nanotubes, silver, antibacterial properties, electrical properties

## Abstract

In the present study, flexible and conductive nanofiber membranes were prepared by coating PLA nanofibrous scaffolds with carbon nanotubes and silver nanoparticles. The morphology and structure of the prepared membrane was characterized, as well as its mechanical properties, electrical sensing behavior during consecutive stretching-releasing cycles and human motion detecting performance. Furthermore, the antibacterial properties of the membrane was also investigated. Due to the synergistic and interconnected three-dimensional (3D) conductive networks, formed by carbon nanotubes and silver nanoparticles, the membrane exhibited repeatable and durable strain-dependent sensitivity. Further, the prepared membrane could accurately detect the motions of different body parts. Accompanied with promising antibacterial properties and washing fastness, the prepared flexible and conductive membrane provides great application potential as a wearable fabric for real-time body motion sensing.

## 1. Introduction

Nowadays, wearable electronics have received tremendous interest in the smart textiles field with the explosive development of information technology, artificial intelligence, electronic technology, etc. [1]. Promising wearable electronics are flexible and conformable, and are able to sense or monitor body conditions, or provide more specific functions, such as information transmission and energy storage [2,3]. Amongst the key components of the wearable electronic system, flexible and conductive fiber (FCB) is extremely significant. To adapt the body movements of humans, the FCB needs to retain electrical stability and maintain its mechanical performance even when it is deformed, bended, or stretched to different shapes [4,5]. Due to this reason, in many recent studies, more and more researchers utilize common-used textile polymers to prepare FCB by incorporating conductive nanoparticles with the textile polymers [6,7]. By such means, the prepared FCB can integrate the flexibility and conformability of the textile polymer and the high conductivity of the nanoparticles. Poly (lactic acid) (PLA), an aliphatic polyester polymer, is the first natural-based synthetic fiber which can be 100% prepared from renewable resources such as corns [8,9]. As a promising textile polymer, PLA owns the merits of low cost, easy processing, dyeing and finishing, and good body conformability [10]. More importantly, this bio-renewable and biodegradable polymer is readily spun into fibers with various spinning methods, including melt spinning, wet spinning, dry spinning, and electrospinning, etc. [11]. Having these advantages, many researchers have focused on the development of FCB using PLA as the polymer substrate in recent years [12,13].

On the other hand, the carbon nanotubes (CNTs) have become a popular nanoparticle for the preparation of conductive polymer composites, in the past few decades, due to their excellent mechanical, thermal, especially electrical properties [14]. A generally accepted approach in preparing CNTs, which incorporate conductive polymer fiber composite, is to directly add the CNTs into the polymer solution before the polymer is spun to fibers [15]. However, the ease of aggregation of the CNTs bundles in polymer matrix might severely deteriorate the inherent properties of the prepared fiber composite [16]. Recently, some researchers prepare conductive fibers by directly coating the conductive nanoparticles onto the surface of the polymer fibers [17]. Meanwhile, many studies have shown that the integration of 0D conductive nanoparticles with 1D CNTs in the polymer matrix could significantly enhance the electrical properties of the prepared polymer composites, since the nanoparticles and the CNTs could form a stable and continuous interpenetrating conductive network in, or on, the polymer [18]. Amongst the 0D nanoparticles, the silver nanoparticles (AgNPs) are a promising candidate, since the AgNPs not only have high electrical conductivity and good oxidation resistance, but also endow the polymer fiber with antibacterial capability, which is also a significant property when designing a wearable material [19,20].

Thus, in the present study, the PLA nanofibrous scaffolds were first prepared by electrospinning. The conductive nanofibrous composite membrane was then prepared by coating multi-walled CNTs (MWCNTs) and AgNPs onto the surface of PLA nanofibrous scaffolds. The dispersion state of MWCNTs and AgNPs on the PLA surface was characterized, and the mechanical and electrical properties of the prepared composite membrane were studied. The impact of cycling stretching-releasing and different body motions on the electrical response of the prepared flexible and conductive membrane were systematically studied. Moreover, the antibacterial performance of the prepared membrane was also investigated.

## 2. Materials and Methods

### 2.1. Materials

PLA (6051D, Natureworks) was purchased from NatureWorks, Minnetonka, MN, USA. MWCNTs were purchased from Shenzhen Nanotech Port Co., Ltd. (China), which had diameters between 60 and 100 nm, length of 15 μm, and purity over 95%. Silver nitrate (AgNO_3_, 99.8%) was purchased from Aladdin Chemical Co., Ltd. (China). Other reagents and solvents were all of analytical grade and used without further purification.

### 2.2. Preparation of PLA Nanofibrous Scaffolds

The PLA nanofibrous scaffolds were prepared using an electrospinning method, described as follows. Typically, 1.5 g of PLA pellets were first dissolved in 10 mL of dimethyl formamide (DMF)/tetrahydrofuran (THF) mixed solution in which the volume ratio of DMF/THF was 1/9. After the PLA was fully dissolved, the PLA solution was then transferred into a 20 mL syringe, equipped with a constant speed syringe pump. For the electrospinning process, a constant distance of 20 cm between the syringe tip and rotating collector was applied, during which the jet rate of the PLA solution was set at 1.5 mL/h, and the applied voltage was set at 20.0 kV. After electrospinning, the PLA nanofibrous scaffolds were maintained at 60 °C for 24 h in a vacuum oven before further processing.

### 2.3. Preparation of MWCNTs/Ag/PLA Nanofibrous Membrane (MAPNM)

**Figure 1:**
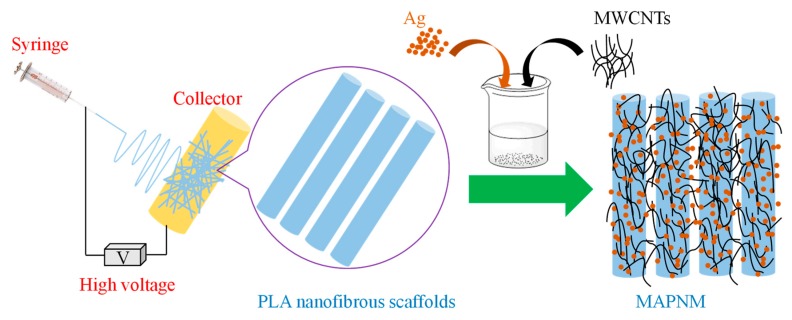
Schematic illustration of the preparation of MWCNTs/Ag/PLA Nanofibrous Membrane (MAPNM).

The MWCNTs were first pre-acidified by concentrated sulfuric acid and nitric acid, according to the previous study. At the same time, 50 mg of AgNO_3_ was dissolved in 50 mL of distilled water (H_2_O). Afterwards, 100 mg of acidified MWCNTs was added into the above AgNO_3_ solution, and the mixture was sonicated for 2 h to obtain a homogeneous dispersion. Above-dried PLA nanofibrous scaffolds in the amount of 0.5 g were then put into the MWCNTs/Ag dispersion. After being soaked in this dispersion for 1 h with continuous magnetic stirring (200 r/min), the coated PLA nanofibrous scaffolds were separated, washed with H_2_O for 3 times to wash the uncoated MWCNTs and AgNO_3_ residues, dried at 60 °C for 24 h in a vacuum oven, and the MWCNTs/Ag/PLA nanofibrous membrane (MAPNM) was finally obtained. The procedures were schematically shown in Figure 1. For comparison, the pure acidified MWCNTs coated PLA nanofirous membrane (MPNM) without the introduction of AgNO_3_ was also prepared following similar preparation procedures.

### 2.4. Characterizations

Scanning electron microscopy (SEM) was conducted by the FEI Quanta 200 (Thermo Fisher Scientific, Hillsboro, USA). Transmission electron microscopy (TEM) images were recorded using a FEI F20 TEM instrument (Thermo Fisher Scientific, Hillsboro, USA). The X-ray diffraction (XRD) was conducted using a Rigaku Smartlab XRD instrument (Rigaku Corporation, Tokyo, Japan) with the Cu Kα radiation source (1.54 Å). Fourier transform infrared spectra (FT-IR) were recorded using a Bruker Vertex80v spectrophotometer (Bruker Optics, Inc., Ettlingen, Germany) with a resolution of 4 cm^−1^ and 16 scans.

### 2.5. Mechanical and Electrical Tests of the Nanofibrous Membrane

The mechanical properties of the membranes were measured by a universal tensile testing machine (Instron 5966, Instron Corporation, Canton, OH, USA) with a 500 N cell at room temperature. The membranes were cut into a 30 × 5 mm^2^ rectangular shape with the thickness of ~0.4 mm. The extension rate was 5 mm/min and the gauge length was 20 mm. The resistance of the prepared nanofibrous membrane was tested by a universal multimeter (Fluke, F115C, Fluke Corporation, Everett, WA, USA). The resistance change of the MAPNM during the stress-strain test was conducted by the Instron 5966 universal testing machine and recorded by the Keithley 2000E multimeter (Tektronix, Inc, Beaverton, OR, USA). The resistance data of the samples was acquired simultaneously as a function of the applied strain. Two conductive silver wires connecting to the multimeter were linked to each end of the samples before testing.

The washing fastness of the MAPNM was conducted by measuring the electrical resistance change (R/R_0_) and mechanical strength of one piece of 30 × 5 × 0.4 mm^3^ MAPNM sample after 1–5 washing cycles. In each washing cycle, the sample was put into 100 mL of 40 °C H_2_O, stirred mechanically at 600 r/min for 20 min, taken out and dried at 60 °C.

### 2.6. Strain Sensing and Motion Sensing Tests of the Nanofibrous Membrane

The resistance change of MAPNM during consecutive stretching/release cycles was also recorded by the Instron 5966 and Keithley 2000E. The sample was stretched to 3% of elongation and released to initial state for 100 cycles. The shape of the MAPNM, gauge length were in accordance with the stress-strain test. The extension speed was set at 40 mm/min. The resistance changes during consecutive body motions, including finger, inner elbow, knee joints and forehead, with different bending angles of the MAPNM were also recorded by Keithley 2000E. During the test, the MAPNM sample, with the shape based on the dimensions, 30 × 5 × 0.4 mm^3^, was tightly attached to the respect part, and each part was bended and released to initial state for 50 time.

### 2.7. Antibacterial Test

The antibacterial properties of the prepared MAPNM and MPNM membranes against Escherichia coli (*E. coli*) and Staphylococcus aureus (*S. aureus*) were evaluated by both inhibition zone and co-culture methods, and described as follows [21]. First, 100 μL of *E. coli* (ATCC8739) or *S. aureus* (ATCC6538) mother liquor was first grown in 900μL of fluid nutrient medium, and cultivated at 37 °C for 12 h to obtain a saturated bacterial suspension of 10^8^–10^9^ CFU/mL. The bacterial suspension was then diluted to 10^5^–10^6^ CFU/mL. For inhibition zone method, the diluted bacterial suspension was coated evenly on the surface of the autoclaved culture dishes. After the prepared membranes (10 × 10 mm^2^) were placed on each bacteria suspension surface, the dishes were then incubated at 37 °C for 18 h. The inhibition zone around each sample was measured for the evaluation of the antibacterial properties of the prepared membranes. For co-culture method, 100 μL of diluted bacterial suspension, 900 μL of bacteria-free phosphate buffer saline solution and 5 mg of smashed membrane sample were mixed together and shock co-cultured at 37 °C for 12 h. The co-cultured suspension was then coated evenly on the surface of the autoclaved culture dishes, and cultivated at 37 °C for another 24 h. The antibacterial properties of the prepared membranes were evaluated by observing the growing status of the bacteria on the culture dish. For comparison, the co-culture of the bacteria, without the incorporation of the prepared membrane samples, was also conducted.

## 3. Results and Discussion

**Figure 2:**
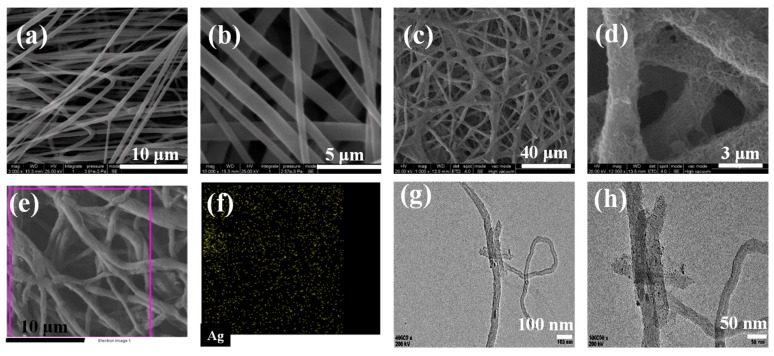
(**a**) Scanning electron microscopy (SEM) image and (**b**) magnified SEM image of Poly (lactic acid) (PLA) nanofibrous scaffolds, (**c**) SEM image and (**d**) magnified SEM image, (**e**) selected region and (**f**) Ag distribution EDS mapping, (**g**) TEM image, and (**h**) magnified TEM image of MAPNM.

The morphology of the MAPNM was investigated first in terms of SEM and TEM shown in Figure 2. It could be seen from Figure 2a,b that the PLA nanofibrous scaffolds had a hierarchical 3D network structure in which the nanofibers interconnect with each other. Moreover, the PLA nanofibers had a smooth and uniform appearance with an average diameter of ~600 nm. After MWCNTs and AgNO_3_ were coated, it was clearly observed from Figure 2c that the MAPNM had a rough nanofiber surface. From the magnified SEM image shown in Figure 2d, it was seen that the MWCNTs were tightly anchored on the surface of the PLA nanofiber surface, which constructed a continuous and integrated conductive pathway. It was notable that the existence of AgNPs could not be observed visually from the SEM image since the AgNPs were rather finite in size. Thus, the energy dispersive spectrum (EDS) mapping of the Ag element was then conducted with the results shown in Figure 2e,f. From the selected region, it could be clearly observed that the AgNPs were sufficiently and uniformly dispersed on the MAPNM surface. The distribution of AgNPs in MAPNM could be also be directly observed through TEM, with the results shown in Figure 2g,h. It could be seen From Figure 2g that the PLA nanofibers entangled with each other, in which fiber-shaped MWCNTs and AgNPs could be observed. From the magnified TEM image shown in Figure 2h, it could be clearly observed that the nanosized AgNPs were uniformly attached on the PLA nanofiber surface. Both SEM and TEM images indicated a uniform and integrated dispersion of MWCNTs and AgNPs on the PLA nanofiber surface.

**Figure 3:**
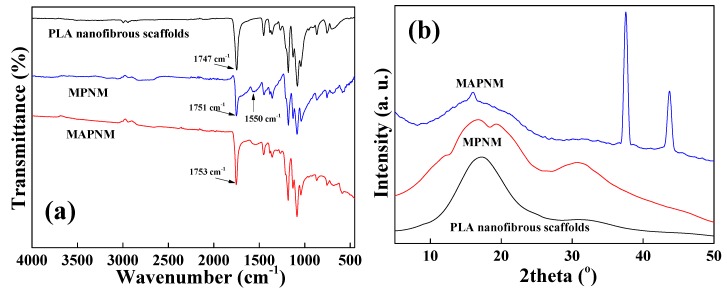
(**a**) FT-IR spectra and (**b**) X-ray diffraction (XRD) patterns of PLA nanofibrous scaffolds, MPNM and MAPNM.

The structural information of the MAPNM was further investigated by FT-IR and XRD. Figure 3a shows the FT-IR spectra of PLA nanofibrous scaffolds, MPNM and MAPNM. It was seen that pure PLA had a characteristic peak in 1747 cm^−1^, which was ascribed to the stretching vibration of its C=O ester groups within PLA skeleton [22]. When the MWCNTs and AgNPs was adhere onto the PLA matrix, this peak in both MPNM and MAPNM shifted a little to 1751 and 1753 cm^−1^, which was might because the oxygen containing groups in acidified MWCNTs interacted with the groups in PLA through hydrogen bonding and/or ionic bonding [23]. Moreover, a series of new peaks at ~1550 cm^−1^ appeared in MPNM and MAPNM, which was ascribed to the vibrations of the benzene rings in MWCNTs [24].

Figure 3b shows the XRD patterns of PLA nanofibrous scaffolds, MPNM and MAPNM. It was observed that PLA had two characteristic peaks located at ~17° and 30°, which were ascribed to the α phase, and α’ phase, of the PLA matrix, respectively [23]. When MWCNTs and AgNPs were coated onto the PLA surface, the characteristic peaks of MWCNTs at ~24° for MPNM and AgNPs at 37° and 43° for MAPNM could be observed from their XRD patterns [25]. For both MPNM and MAPNM, these two peaks could also be observed, which became broader and weaker, thereby, indicating a lower degree of crystallinity of the PLA in the composites. This was ascribed to the incorporation of the conductive nanomaterials, which restrained the mobility and affected the regularity of the polymer chain [26]. These two peaks shifted into smaller 2θ values, indicating the formation of interactions between the nanoparticles and matrix, which was in accordance with the FT-IR results.

**Figure 4:**
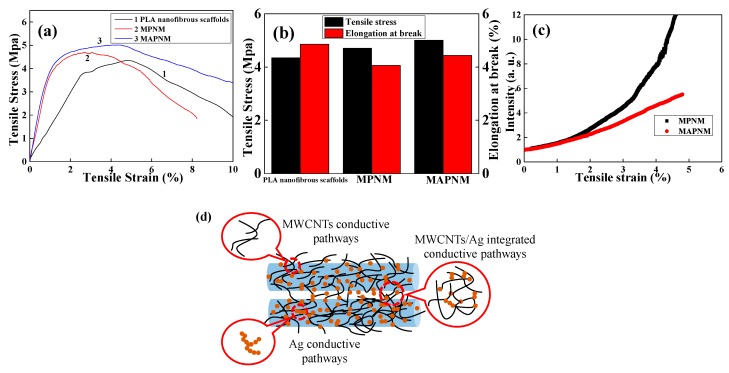
(**a**) Stress-strain curves and (**b**) tensile stress and strain values of PLA nanofibrous scaffolds, MPNM and MAPNM, (**c**) electrical resistance response of MPNM and MAPNM during stress-strain test, (**d**) schematic illustration of the integrated 3D conductive network within MAPNM.

The mechanical properties of the prepared samples were then studies through stress-strain test with the results shown in Figure 4a. Figure 4b shows the detailed mechanical stress and strain values of the respect sample. As could be seen, the PLA nanofibrous scaffolds showed a classic plastic mechanical behavior with the tensile stress of 4.35 MPa, and breaking elongation of 4.86%. After the conductive nanoparticles were coated on the surfaces of the PLA nanofibers, both MPNM and MAPNM showed an obvious increase in the tensile stress and a decrease in the elongation at break. Specifically, the tensile stress and tensile strain for MAPNM were 5.01 MPa, and 4.44%, respectively. This was ascribed to the interactions between the rigid nanoparticles and the PLA matrix, which resulted in the mechanical strength enhancement of the composites compared with the pure PLA membrane.

The electrical response of the MPNM and MAPNM, during the stress-strain test, were also studied. Figure 4c shows the relative resistance change of the MPNM and MAPNM as a function of the applied strain. It was clearly observed that, due to the excellent electrical conductivity of the MWCNTs and the AgNPs, both MPNM and MAPNM exhibited promising electrical properties, which showed a monotonic increase in their R/R_0_ values, and preserved their electrical properties until the membranes were stretched to break. Moreover, it was also seen that during the whole stretching test process, the MAPNM exhibited a near-linear response to the applied strain. On the contrary, the MPNM showed more drastic change in its resistance during the stress-strain test. It is generally known that the resistance of the conductive polymer composite increases with the increase of the applied strain, which is ascribed to the disconnection of the conductive pathways while the polymer composite is stretched [27]. Many previous studies have shown that, when designing a conductive polymer composite, the incorporation of multi-dimensional conductive particles composed of one 0D component and one 1D component could form synergetic and well-connected conductive pathways within the polymer matrix, through the confinement effect and volume exclusion effect, leading to much stronger 3D conductive networks of the prepared polymer composite [28,29]. As schematically shown in Figure 4d, for MAPNM prepared in the present study, the hybrid MWCNTs/AgNPs formed synergistic and interconnected networks on the PLA nanofiber surfaces which provided 3D conductive pathways during the MAPNM was stretched, resulting a linear response of its resistance to the applied strain.

**Figure 5:**
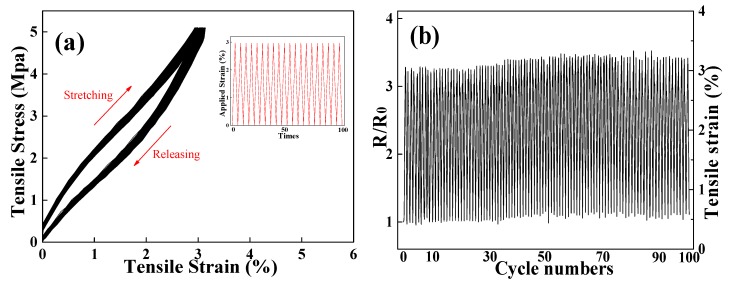
(**a**) Cyclic stress-strain curves and (**b**) corresponding resistance response of MAPNM from 0 to 3% for 100 cycles.

For a strain sensor, the longtime-use stability and conductive reconstruction after the applied mechanical deformation is released are two vital factors to be considered. On this basis, the cyclic mechanical strength and electrical response were investigated. Figure 5 shows 100 stress-strain cycles of the MAPNM with the strain of 3%, and the resistance response of the MAPNM during the 100 cycles. As was observed in Figure 5a, the MAPNM could recover to its initial point quickly with relatively stable hysteresis loop when the applied mechanical stimulus was removed, indicating a promising mechanical stability and durability of the prepared MAPNM [30]. It was also observed from Figure 5b, which recorded the dynamic resistance change during 100 stress-strain cycles that the R/R_0_ responded simultaneously from 1.0 to 3.3 with the applied strain. Moreover, the R/R_0_ barely changed, even after 100 cycles of stretching-releasing times, which indicated high cyclic repeatability and stability.

**Figure 6:**
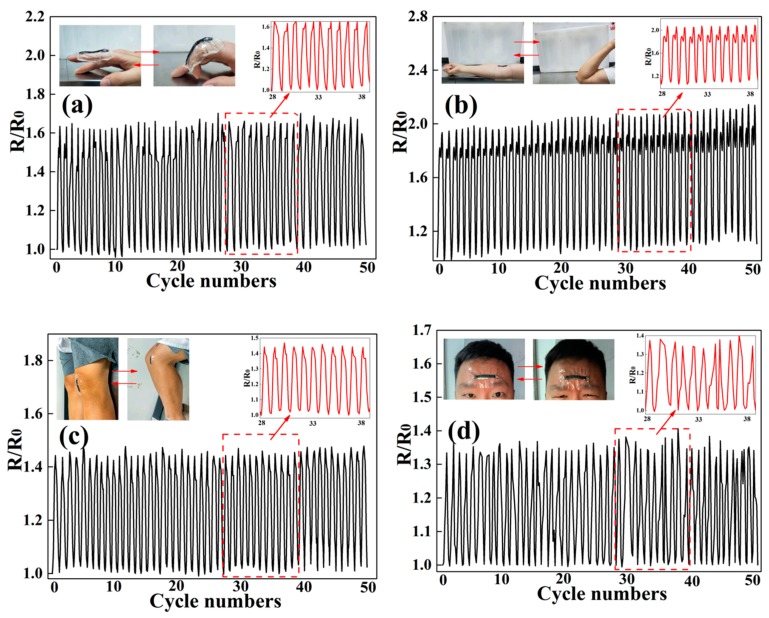
Real-time electrical resistance response of MAPNM for (**a**) finger bending, (**b**) inner elbow bending, (**c**) knee bending and (**d**) forehead movement for 50 cycles.

To further prove the application potential of the prepared MAPNM as the wearable strain sensor for use in real-life body detection, the MAPNM was adhered to different body parts, including finger joint, inner elbow, knee, and forehead to detect the real-time body motion signals of these parts. Figure 6 shows the resistance change signals as a function of the folding and unfolding of the motions of the above-mentioned body parts for 50 consecutive cycles. As can be seen, for all conducted body motions, the MAPNM could respond rapidly, accurately, and consistently. For example, it could be seen from Figure 6a that, when the finger folded and unfolded repeatedly, the R/R_0_ of the MAPNM increased simultaneously and periodically with the finger folding, and decreased to its original resistance value when the finger was unfolded. For all 50 folding-unfolding cycles, the R/R_0_ of the MAPNM was able to keep its value from 1.0 to 1.62. Similar responses of the MAPNM could be also detected when the MAPNM was attached to inner elbow, knee, and forehead, as shown in Figure 6b–d. As a result, the prepared MAPNM had promising potentials as the real-time body motion sensing detector.

**Figure 7:**
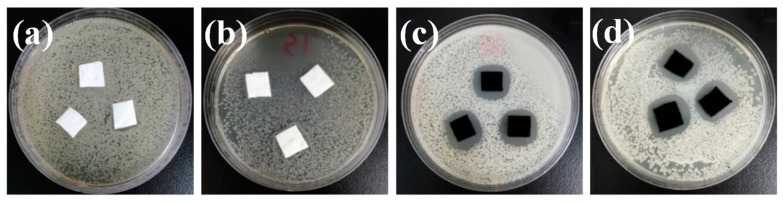
Antibacterial inhibition zone images of PLA nanofibrous scaffolds against (**a**) *E. coli* and (**b**) *S. aureus*, and MAPNM against (**c**) *E. coli* and (**d**) *S. aureus.*

The antibacterial properties are also significant to a wearable strain sensor, given the strain sensor is always exposed to daily-life environments, encountered frequently with various bacteria. Thus, the *E. coli* which represented the Gram-negative bacteria, and *S. aureus* which represented the Gram-positive bacteria, were employed to investigate the antimicrobial performance of the prepared nanofibrous membranes. Figure 7 shows the growth of inhibition zones of E. coli and S. aureus treated with PLA nanofibrous scaffolds and MAPNM for 18 h at 37 °C. As was shown, the PLA nanofibrous scaffolds did not show any antibacterial effect to both *E. coli* and *S. aureus*. Comparatively, MAPNM had a very promising antibacterial performance whose inhibition mean zones were 0.48 cm against *E. coli*, and 0.45 cm against *S. aureus*, respectively. This was because when MAPNM incubated with the bacteria, the Ag^+^ ions could be released from the MAPNM surface, which were able to kill adjacent bacteria quickly through electrostatic adsorption and protein coagulation [31]. Furthermore, the reason why the inhibition zone of MAPNM against *S. aureus* was smaller than that of *E. coli*, as the Gram-positive had a relatively thicker cell wall with peptidoglycan layers, which were more difficult to penetrate by the Ag+ ions, when compared with Gram-nagative *E. coli* [32].

**Figure 8:**
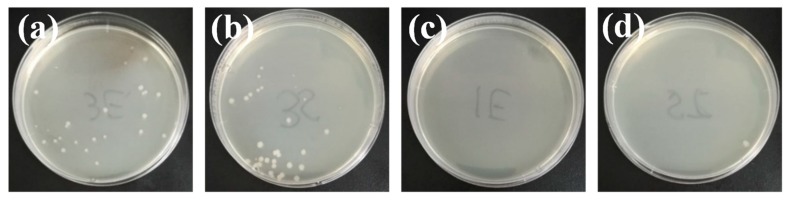
Antibacterial co-culture images PLA nanofibrous scaffolds against (**a**) E. coli, and (**b**) S. aureus, and MAPNM against (**c**) E. coli and (**d**) S. aureus.

The bacteria co-culture approach was also introduced to further prove the antibacterial performance of the prepared MAPNM with the results, as shown in Figure 8. It could be clearly seen that after 24 h of cultivation, still a large amount of *E. coli* and *S. aureus* were able to survive and grow in the presence of the PLA nanofibrous scaffolds. Meanwhile, the MAPNM could kill almost all the bacteria, indicating, again, a promising antibacterial property of the MAPNM against both Gram-positive and Gram-negative bacteria.

**Figure 9:**
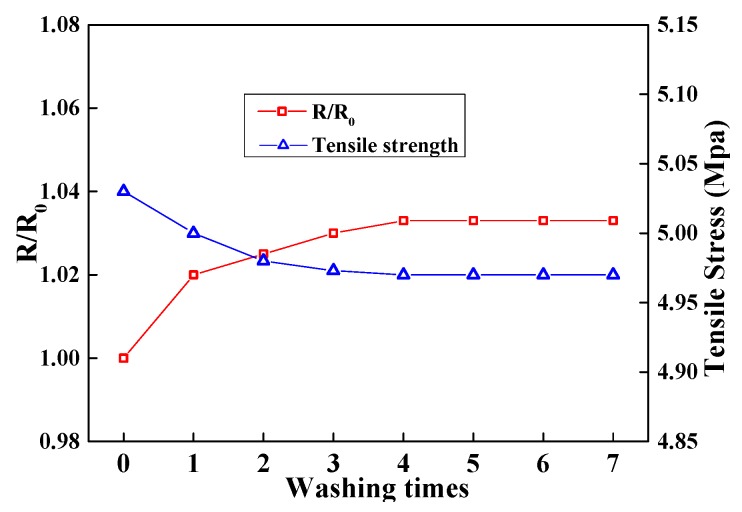
R/R_0_ and mechanical strength of MAPNM for seven washing runs.

As a wearable conductive fabric, property stability after several washing cycles also needs to be taken into consideration. Figure 9 shows the washing speed of the MAPNM for 7 washing cycles. It could be seen that after the MAPNM was washed for 7 cycles, the tensile stress of the sample decreased a little, which was because a small number of conductive nanoparticles were removed in the initial washing cycles. After 4 washing cycles, both the R/R_0_ and tensile stress became relatively stable. This was because most of the conductive nanoparticles adhered to the PLA matrix tightly, with strong interactions through hydrogen bonding and/or ionic bonding, which was in accordance with the FT-IR and XRD results.

## 4. Conclusions

To conclude, we prepared conductive and antibacterial MAPNM, using PLA nanofibrous scaffolds, coated with MWNCTs and AgNPs. The MWCNTs and AgNPs was attached on the PLA nanofiber surface tightly with strong interactions. The incorporation of the MWCNTs and AgNPs obviously enhanced the mechanical properties of the prepared MAPNM. Moreover, since the hybrid conductive nanoparticles integrated by MWCNTs, and AgNPs formed a 3D network with abundant conductive pathways, the MAPNM showed a quick and accurate electrical response to the applied strain during the stress-strain test. In addition, the MAPNM exhibited promising stability and repeatability in its mechanical strength and electrical response after 100 times of consecutive stretching-releasing cycles. The MAPNM could be further utilized to precisely detect the human motions of different body parts like finger, elbow, knee, and forehead. Furthermore, the MAPNM showed promising antibacterial properties to both Gram-negative *E. coli* and Gram-positive *S. aureus*. Further, the MAPNM showed good washing speed, which could preserve its properties after several times of washing. The MAPNM prepared in the present study showed great promise as the wearable conductive fabric to monitor the human activities in real life.
